# The top-down approach to measurement uncertainty: which formula should we use in laboratory medicine?

**DOI:** 10.11613/BM.2020.020101

**Published:** 2020-04-15

**Authors:** Flávia Martinello, Nada Snoj, Milan Skitek, Aleš Jerin

**Affiliations:** 1 Department of Clinical Analysis, Federal University of Santa Catarina, Florianopolis, Brazil; 2 Institute of Clinical Chemistry and Biochemistry, University Medical Centre Ljubljana, Ljubljana, Slovenia

**Keywords:** measurement uncertainty, bias, uncertainty, total error, quality control

## Abstract

**Introduction:**

By quantifying the measurement uncertainty (MU), both the laboratory and the physician can have an objective estimate of the results’ quality. There is significant flexibility on how to determine the MU in laboratory medicine and different approaches have been proposed by Nordtest, Eurolab and Cofrac to obtain the data and apply them in formulas. The purpose of this study is to compare three different top-down approaches for the estimation of the MU and to suggest which of these approaches could be the most suitable choice for routine use in clinical laboratories.

**Materials and methods:**

Imprecision and bias of the methods were considered as components of the MU. The bias was obtained from certified reference calibrators (CRC), proficiency tests (PT), and inter-laboratory internal quality control scheme (IQCS) programs. The bias uncertainty, the combined and the expanded uncertainty were estimated using the Nordtest, Eurolab and Cofrac approaches.

**Results:**

Using different approaches, the expanded uncertainty estimates ranged from 18.9-40.4%, 18.2-22.8%, 9.3-20.9%, and 7.1-18.6% for cancer antigen (CA) 19-9, testosterone, alkaline phosphatase (ALP), and creatinine, respectively. Permissible values for MU and total error ranged from 16.0-46.1%, 13.1-21.6%, 10.7-26.2%, and 7.5-17.3%, respectively.

**Conclusion:**

The bias was highest using PT, followed by CRC and IQCS data, which were similar. The Cofrac approach showed the highest uncertainties, followed by Eurolab and Nordtest. However, the Eurolab approach requires additional measurements to obtain uncertainty data. In summary, the Nordtest approach using IQCS data was therefore found to be the most practical formula.

## Introduction

Uncertainty is a parameter associated with the result of a measurement that characterizes the dispersion of the values that could reasonably be attributed to the measurand (*1*). By quantifying the variation in the results, both the clinical laboratory performing the measurements and the physician receiving the results can have an objective estimate of the quality of the results (*2*).

The measurement uncertainty matters in laboratory medicine to define the test suitability, to verify quality of *in vitro* diagnostics products, to provide evidence of unpredictable bias and to demonstrate the test clinical suitability (*3*). In addition, clinical laboratories looking forward the accreditation under the ISO 15189 standard, shall determine measurement uncertainty for each measurement procedure, and define and regularly check their performance requirements concerning uncertainty (*4*). The International Organisation for Standardisation (ISO) standard 15189 does not suggest any particular approach for determining measurement uncertainty (MU) (*5*). It states, “The laboratory shall determine measurement uncertainty for each measurement procedure…”, thus allowing significant flexibility on how to determine it (*4*, *6*).

The traditional methods for estimating the MU are described in the Guide to the expression of uncertainty in measurement (GUM). The main problems with the GUM approach for medical laboratory personnel is its reliance on complex statistical procedures and the fact that some error sources require derivative functions, which are not always estimable (*5*).

The “bottom-up” approach aims to estimate the individual contribution of every step of the process to the overall uncertainty (*7*). Briefly, it is based on all conceivable sources of uncertainty that must be systematically evaluated, and demands a clear description of what is being measured, including the relationship between the quantity and the parameters upon which it depends. Then the identified uncertainties are combined to generate a combined uncertainty of the result using statistical propagation rules (*8*). This exhaustive approach can be time-consuming to apply to laboratory medicine tests in terms of designing and performing experiments to provide additional data for the estimation. It does, however, enable the analyst to identify critical stages in the method and is useful for method optimization or troubleshooting during development (*7*).

The “top-down” approach directly estimates the measurement uncertainty typically by evaluating quality control (QC) data or method verification experiment data (*9*). The “top-down” approach is more practical and cost-effective, can be updated as further data becomes available through results from routine internal quality control (IQC) and proficiency tests (PT). More importantly, no statistically significant differences have been found between the uncertainty values obtained by either approach (*7*, *9*). Although a study with practical and detailed examples of all the two approaches is hard to find (*9*).

With the “top-down” approach, the MU should include both the imprecision and bias component if the latter is considered significant. Uncertainties arising from random and systematic effects are treated alike. Through the application of the uncertainty propagation principles, the uncertainty contributions are then summed up to yield the so-called combined standard uncertainty (*10*-*13*). Two approaches can be used to assess bias: it can be based on certified reference materials or the results from quality control material procedures, such as PT (*14*). Recently, other kind of inter-laboratory proficiency testing scheme data was proposed to estimate the uncertainty related to the bias: the inter-laboratory internal quality control scheme (IQCS) (*8*).

The principal guidelines from various bodies (Nordtest, Eurolab and Cofrac) all propose different approaches for calculating measurement uncertainty. Handbook for calculation of measurement uncertainty in environmental laboratories (Nordtest) calculated measurement uncertainty on the basis of the within-laboratory reproducibility and the uncertainty of laboratory bias, which is estimated from certified reference material, inter-laboratory comparisons, or recoveries (*15*, *16*). European Federation of National Associations of Measurement, Testing, and Analytical Laboratories (Eurolab) based the measurement uncertainty calculus on the dispersion of the relative difference of the results given by a laboratory on different PT schemes (11,16))). French accreditation body (Cofrac) suggests a different method based on data from combined data from IQC and calibration uncertainty (*17*, *18*).

Despite the large amount of data available on MU, information about the practicality of the formulas in laboratory routines is scarce. In this context, it is important to assess their feasibility with the aim of being able to select one that will be reliable and adequate for each laboratory method.

As the uncertainties of the preanalytical phase are not established enough for laboratory medicine tests to apply the “bottom-up” approach, we selected some clinical chemistry tests to serve as examples to compare different practical “top-down” approaches for estimating MU, considering the imprecision and bias of the methods as components with similar statistical properties. The purpose of this study is to compare three different top-down approaches for the estimation of the MU and to suggest which of these approaches could be the most suitable choice for routine use in clinical laboratories.

## Materials and methods

This is a retrospective diagnostic accuracy study developed at the Institute of Clinical Chemistry and Biochemistry of the University Medical Centre Ljubljana, Ljubljana, Slovenia.

The MU was established for the four following laboratory tests to evaluate and compare different manners of estimation: creatinine, alkaline phosphatase (ALP) (Advia 1800, Siemens, Tarrytown, USA), testosterone (Cobas e 411, Roche Diagnostics, Mannheim, Germany), and cancer antigen (CA) 19-9 (Architect i1000, Abbott Diagnostics, Abbott Park, USA). These tests are typical of enzymes, tumour markers, biochemistry and hormones laboratory tests groups.

The allowable total error and the permissible uncertainty were considered to represent the uncertainty target (*5*, *19*).

The permissible uncertainty estimate was based on a non-linear relationship between biological and analytical variation, as proposed by Haeckel *et al.* (*19*). The calculation steps were performed using the reference limits (RL) for the adult males in our laboratory (creatinine 44-97 µmol/L, ALP < 128 U/L, CA 19-9 < 37 kU/L, testosterone 8.8-30.6 nmol/L). In cases where a lower RL was unknown, it was set at 15% of the upper RL (*19*).

The well-established allowable total error was estimated according to Westgard, considering the desirable goals based on biological variation with a 95% confidence level (*20*-*22*).

Imprecision and bias uncertainty was considered components of MU, and they are represented as the square roots of the variances in their respective estimators. They were then combined to produce a bias uncertainty estimate. The uncertainty estimates were performed and presented as relative uncertainties (%), which permits their comparison and application over a range of values.

### Imprecision (CV_WL_)

Internal quality control data were collected from January 2016 to July 2018. The control materials for ALP and creatinine were from Biorad (Hercules, USA), for CA 19-9 from Technopath (Tipperary, Ireland) and for testosterone from Roche Diagnostics (Mannheim, Germany). The intermediate precision was determined as the long term, within-laboratory coefficient of variation for each concentration level for at least 235 IQC results. The arithmetic average of the within-laboratory coefficient of variation found for each level was taken as the imprecision (CV_WL_).

### Bias

The bias calculation was performed from three data sources: certified reference calibrators (CRC), PT, and inter-laboratory internal quality control scheme (IQCS) (*8*, *23*).

#### Bias from certified reference calibrators

Commercial calibrators different from those used for calibration of the test, were used as CRCs. According to the manufacturers, calibrator’s assigned values and respective expanded (k = 2) uncertainties were: ALP_2c calibrator traceable to IFCC (530 ± 4 U/L) and CrRE_2c calibrator traceable to IDMS Reference Method/NIST SRM 967 (724.9 ± 11.6 µmol/L) from Siemens (USA); CA 19-9 calibrators traceable to International Reference Standard (30 ± 0.51 kU/L, 250 ± 2.04 kU/L, 1200 ± 19.86 kU/L) from Abbott (USA), testosterone calibrators traceable to ID-GC/MS (1.32 ± 0.04 nmol/L, 43.03 ± 1.31 nmol/L) from Roche (Germany).

The measurement of one (creatinine and ALP), two (testosterone), or three (CA 19-9) CRCs were performed in 5 to 17 different analytical series, and the results were used to estimate the bias through equation (Eq.) 1 (*15*):



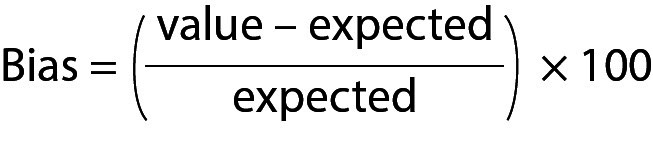



When more than one CRC was used, the root mean square of the individual bias values (RMS_bias_) was calculated according to Eq. 2 (*15*), where *n* is the number of CRCs used.



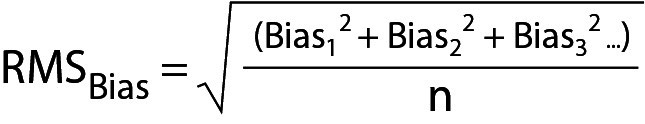



#### Bias from proficiency tests

The external quality control data were collected from February 2013 to May 2018. Data for ALP, creatinine and CA 19-9 were obtained from Instand (Düsseldorf, Germany) and for testosterone from Labquality (Helsinki, Finland) proficiency test.

The bias from PT was calculated using only satisfactory PT results, and results not complying with established PT criteria (*i.e.,* results with z-scores >2 or <−2) were discarded. For each analyte, the results from 13 PT rounds were used for the calculation, with each one involving at least 10 participating laboratories. The bias from each round was calculated by considering the target value obtained from the peer group as the expected value (*via* Eq. 1), while the RMS_bias_ was calculated using Eq. 2, where *n* is the number of PT rounds in this case.

#### Bias from IQCS (Unity program)

The bias was calculated using six IQCS peer-comparison rounds for the creatinine and ALP analytes, each one including at least 49 participating laboratories. The bias from each peer-comparison round was calculated by considering the average value of the peer group as the expected value (again with Eq. 1), while the RMS_bias_ was calculated according to Eq. 2, where *n* is now the number of IQCS peer-comparison rounds.

### Bias uncertainty (b)

Estimating the bias uncertainty, u(Bias), was achieved through three formulas:

1. The Nordtest approach using the bias from PT, CRC, and IQCS (*15*).
2. The Eurolab approach using the bias from CRC and PT (*11*). Bias uncertainty was not calculated using IQCS data due to absence of duplicate results as required.
3. The Cofrac approach using the bias from PT and IQCS (*17*). Bias uncertainty was not calculated using CRC data because the Cofrac MU formula does not include it on the uncertainty estimate.

#### Nordtest approach

The uncertainty of proficiency test, u(PT), for each analyte was calculated through Eq. 3.
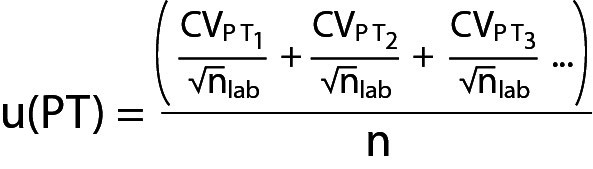
where CV_PT_ is the CV of each PT round and n_lab_ is the number of participating labs in each round, while n is the number of PT rounds.

The u(Bias_PT_) was calculated as shown in Eq. 4.



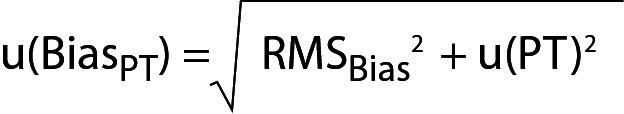



The uncertainty of CRC, u(CRC), for each analyte was calculated as shown in Eq. 5:
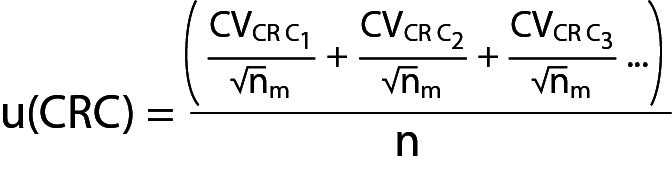
where CV_CRC_ is the CV among the CRC measurements for each analytical series, n_m_ is the number of CRC measurements, and n is the number of CRCs used.

The u(Bias_CRC_) was calculated as shown in Eq 6:
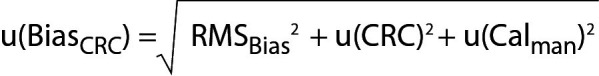
where *u(Cal_man_)* is the calibrator expanded uncertainty provided by the manufacturer divided by the coverage factor k, which was considered to be 2 in our study (*11*, *15*).

The uncertainty of IQCS, u(IQCS), was calculated as shown in Eq. 7:
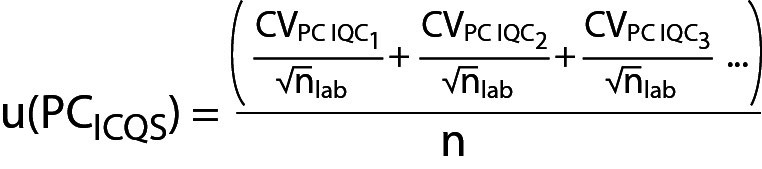
where CV_PCIQCS_ is the CV of each monthly peer-comparison bias from the IQCS report, n_lab_ is the number of participating laboratories in the IQCS peer-comparison reports, and *n* is the number of IQCS peer-comparisons.

The u(Bias_PCIQCS_) was calculated as shown in Eq. 8,



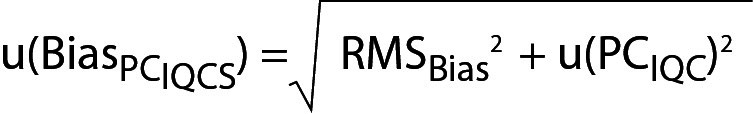



#### Eurolab approach

According to the Eurolab Technical Report the bias contribution to measurement uncertainty is obtained from the mean deviation, the uncertainty of the target value, and the imprecision of the mean value of the replicated measurements performed in the bias investigation, as shown in Eq. 9 (*5*):
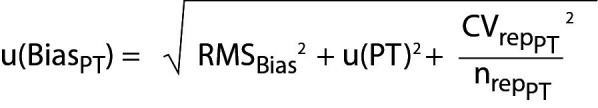
where CV_repPT_ is the CV among the replicated PT measurements and n_repPT_ is the number of replications.

This formula can also be applied using u(PC_IQCS_), the CV among the replications of IQCS measurements (CV_repIQCS_), and the number of replications for the IQC (n_repIQCS_) instead of u(PT), CV_repPT_, and n_repPT_, respectively. Eurolab and Nordtest have the same approach for calculating the uncertainty of the CRC bias (*11*, *15*).

#### Cofrac approach

According to the Cofrac SH GTA 14 document the uncertainty can be evaluated based on the external evaluations’ uncertainty, which is obtained from the deviation of the uniform distribution law (divide the half-range by the square root of 3) and the coefficient of variation for the bias, as shown in Eq. 10 (*17*).
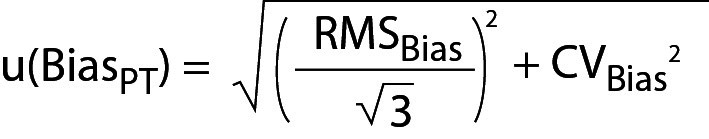
where CV_Bias_ is the CV of the averaged biases from different PT rounds, IQCS comparisons, or CRC materials.

The estimate of uncertainty using CRCs considers just the calibrator uncertainty provided by the manufacturer. As the bias component is not considered in this uncertainty estimate, this approach was not applied in our study.

### Combined uncertainty

Combined uncertainty was estimated according to Eq. 11:



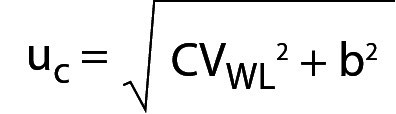



### Expanded uncertainty

The expanded uncertainty U was estimated by applying the coverage factor k = 2 to the combined uncertainty, as shown in Eq. 12:



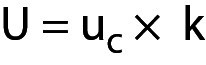



## Results

The results for the bias, bias uncertainty, and imprecision for the selected parameters are presented in Table 1.

**Table 1:** Bias estimated from different data, bias uncertainty calculated according to each approach, within-laboratory coefficient of variation and calibrator uncertainty

**Parameter**				**Bias uncertainty (%)**										
**Bias (%)**			**Nordtest**			**Eurolab**			**Cofrac**			**CV_WL_ (%)**	***u*Cal (%)**
**PT**	**CRC**	**IQCS**	**PT**	**CRC**	**IQCS**	**PT**	**CRC**	**IQCS**	**PT**	**CRC**	**IQCS**		
**CA 19-9**	8.9	2.3	NC	9.1	2.4	NC	9.5	2.4	ND	18.0	NE	NC	9.2	0.7
**Testosterone**	6.2	5.5	NC	6.6	7.9	NC	6.9	7.9	ND	9.5	NE	NC	6.3	1.4
**ALP**	3.6	2.1	3.2	3.9	2.2	3.3	9.6	2.2	ND	4.7	NE	4.9	4.1	0.4
**Creatinine**	5.2	3.6	2.8	5.3	3.7	2.9	8.6	3.7	ND	9.1	NE	4.4	2.0	0.8
CA – cancer antigen. ALP - alkaline phosphatase. PT - proficiency test. CRC - certified reference calibrator. IQCS – inter-laboratory internal quality control scheme. CV_WL_ - imprecision expressed as the within-laboratory coefficient of variation. uCal - calibrator uncertainty provided by the manufacturer. NC - no peer group for comparison. ND - no duplicate results available for IQC. NE - does not include bias for the uncertainty estimate.														

Table 2 presents the MUs obtained through Nordtest, Eurolab, and Cofrac formulas, which incorporate biases achieved through different means. The same table also shows the permissible uncertainty, the analytical total error, and the allowable total error for each test.

**Table 2:** Results for allowable total error, analytical total error, permissible uncertainty, and the expanded uncertainty obtained through three different formulas for each measured parameter

			**Expanded uncertainty (%)**										
			**Nordtest**			**Eurolab**			**Cofrac**				
**Parameter**	**Allowable** %TE*	**%TE**	**PT**	**CRC**	**IQCS**	**PT**	**CRC**	**IQCS**	**PT**	**CRC**	**IQCS**	**Permissible** **%U^†^**	**Permissible** **%U for PT^†^**
CA 19-9	46.1	22.4	25.8	18.9	NC	26.4	18.9	ND	40.4	NE	NC	16.0	26.2
Testosterone	13.6	15.7	18.2	20.1	NC	18.6	20.1	ND	22.8	NE	NC	13.1	21.6
ALP	10.7	9.7	11.4	9.3	10.5	20.9	9.3	ND	12.4	NE	12.8	16.0	26.2
Creatinine	7.5	6.8	11.3	8.4	7.1	17.6	8.4	ND	18.6	NE	9.7	10.6	17.3
CA – cancer antigen. ALP - alkaline phosphatase. TE - total error. U - expanded uncertainty. PT - proficiency test. CRC - certified reference calibrator. IQCS - interlaboratory internal quality control scheme. NC - No peer group for comparison. ND - No duplicate results available for IQC. NE - Does not include bias for the uncertainty estimate. *According to the desirable goals based on biological variation. ^†^According to Haeckel *et al.* (19).													

## Discussion

There is no agreement on how to measure the MU in the clinical laboratory; therefore, laboratory professionals have to decide what data and which formula should be used. Our study showed that calculations using PT data generally gave higher values of bias, bias uncertainty, and expanded uncertainty than using IQCS or CRCs data. Based on our results, it was also clear that the choice of a formula greatly influenced the bias uncertainty and, consequently, the expanded uncertainty. The Cofrac formula gave higher MU results than the Eurolab and Nordtest formulas with the use of either PT or IQCS data, as previously reported (*18*). The reason for such a result might be the use of CV of averaged biases of different rounds of PT or IQCS rather than their uncertainty.

Herein, the MU was not calculated by the IQCS Eurolab approach because it requires a coefficient of variation for the control material results. Indeed, it has not been a common laboratory practice to perform control material analysis in duplicate. Therefore, we considered a disadvantage of the Eurolab approach. Additionally, the MU was also not calculated by the CRC Cofrac approach, since the formula does not include the bias uncertainty when using CRC data. In this context, all results of the present study were based on the imprecision and the bias components of the analytical methods.

Our results showed that bias and its uncertainty contributed similarly to the overall MU, corroborating partly the study of Padoan *et al.* (*24*). However, it has been proposed that the imprecision component of uncertainty includes some bias effects and exerts a greater influence on the expanded uncertainty than the bias (*19*, *23*). Other authors even suggest that harmonization of methods could minimize the bias and, essentially, make both total error and MU a result of imprecision (*5*, *6*). For clinical purposes, it has been suggested that the appropriate choice for estimating MU of laboratory results is influenced by the intended use (*25*). For example, when comparing two consecutive results of the same patient over a short time interval, the imprecision of the analytical method is considered the most relevant MU component; while comparing results with a decision limit or a reference interval, the bias predominates (*23*, *25*). However, it should be noted that is not suitable to have different uncertainties for a single parameter used in two situations, which strengthens the importance of using both components, bias and imprecision, to calculate the MU.

How to calculate the bias component of uncertainty is still a matter of debate. The observed differences between the bias results depended on the source of data (PT, IQCS or CRC) used for calculation. However, calculations using the PT and IQCS data do not take into account the uncertainty accumulated in upper levels of the metrological traceability chain (*23*). Moreover, using data from PT to calculate the bias incurs the risk of overestimating the bias component, probably because some random variation is present in the PT results since tests are usually performed only a few times *per* year (*23*). In our study, the highest bias obtained using PT data, which is in agreement with the results reported by Rigo-Bonnin *et al.* may partly reflect that CRCs data were measured values, while PT values were assigned from the participants’ results (*8*). On the other hand, Ceriotti reported higher bias values using IQCS data (*23*).

However, PT or IQCS data is considered advantageous because of using control materials similar to routine samples (*11*, *18*). Additionally, the IQCS management programs, which perform peer-comparisons of IQC results, can provide automatically calculations in a very efficient manner, which was here considered the most practical method. Taking into account that IQCS perform analyses more frequently, the bias calculated using IQCS data can also be considered more reliable than the bias obtained from different rounds of a PT. Furthermore, compared to CRC data, the use of IQCS perfectly represents the variability of analytical conditions in the laboratory.

Regarding the bias from CRCs, Theodorou *et al.* considered 2.7% of uncertainty of a certified reference material sufficiently small to be ignored in the expanded uncertainty (*10*). In our study, the uncertainty of the CRCs was lower than 2.7% and, consequently, produced the lowest MUs. The Eurolab and Nordtest approaches gave identical results, which was not surprising as they use the same formula to calculate uncertainty using CRC data. It is also important to note that the calibrators’ uncertainties have not been published in their package inserts. Therefore, we had to obtain these values from manufacturers, which is not always easy (*23*).

In addition to different calculations, the interpretation of MU can also be performed using different specifications of analytical quality, *i.e.,* permissible uncertainty, allowable TE, CLIA, RiliBäk, and other PT providers (*26*). The permissible uncertainty limits related to biological variation have often been preferred because of their scientific basis, which can be applied to all methods and it seems to be more rigid than allowable TE (*5*, *19*, *27*). According to Qin *et al.* a relatively high percent of laboratories may not be able to remain within the permissible limits for immunoassays (*28*). In fact, this prediction was true in our study, our results indicated that for immunoassays it is not easy to meet both permissible uncertainty and allowable TE.

In summary, the Cofrac approach tended to overestimate the MU, while the Eurolab approach required additional measurements (duplicates) to obtain uncertainty data. Based on our study, the Nordtest approach using bias from the IQCS can be considered the most practical approach for estimating the MU.

## References

[1] Bureau International des Pois et Mesures. JCGM 200:2012, International vocabulary of metrology – basic and general concepts and associated terms (VIM). Available at: http://www.bipm.org/en/publications/guides/vim.html. Accessed: November 17th 2019.

[2] CLSI. Expression of measurement uncertainty in laboratory medicine; approved guideline. CLSI document EP29-A. Wayne, PA: Clinical and Laboratory Standards Institute, 2012.

[3] Infusino I Panteghini M. Measurement uncertainty: Friend or foe? Clin Biochem. 2018;57:3–6. 10.1016/j.clinbiochem.2018.01.025 29410277

[4] International Organization for Standardization. Third edition, ISO 15189:2012, Medical laboratories – requirements for quality and competence. Geneva, Switzerland, 2012. 10.1111/j.1423-0410.2002.tb05329.x 12617164

[5] Farrance I Badrick T Sikaris KA. Uncertainty in measurement and total error - are they so incompatible? Clin Chem Lab Med. 2016;54:1309–11. 10.1515/cclm-2016-0314 27227711

[6] Farrance I Badrick T Frenkel R. Uncertainty in measurement and total error: different roads to the same quality destination? Clin Chem Lab Med. 2018;56:2010–4. 10.1515/cclm-2018-0421 29949508

[7] Dabalus Islam M Schweikert TM Cannavan A. Comparison of methods for the estimation of measurement uncertainty for an analytical method for sulphonamides. Food Addit Contam. Food Addit Contam Part A Chem Anal Control Expo Risk Assess. 2008;25:1439–50. 10.1080/02652030802189765 19680854

[8] Rigo-Bonnin R Blanco-Font A Canalias F. Different top-down approaches to estimate measurement uncertainty of whole blood tacrolimus mass concentration values. Clin Biochem. 2018;57:56–61. 10.1016/j.clinbiochem.2018.05.005 29750938

[9] Lee JH Choi JH Youn JS Cha YJ Song W Park AJ. Comparison between bottom-up and top-down approaches in the estimation of measurement uncertainty. Clin Chem Lab Med. 2015;53:1025–32. 10.1515/cclm-2014-0801 25539513

[10] Theodorou D Meligotsidou L Karavoltsos S Burnetas A Dassenakis M Scoullos M. Comparison of ISO-GUM and Monte Carlo methods for the evaluation of measurement uncertainty: Application to direct cadmium measurement in water by GFAAS. Talanta. 2011;83:1568–74. 10.1016/j.talanta.2010.11.059 21238753

[11] Eurolab Technical Report N.1/2007. Measurement uncertainty revisited: alternative approaches to uncertainty evaluation, Technical Committee for Quality Assurance in Testing (TCQA), Paris, France, 2007.

[12] Magnusson B Ossowicki H Rienitz O Theodorsson E. Routine internal- and external-quality control data in clinical laboratories for estimating measurement and diagnostic uncertainty using GUM principles. Scand J Clin Lab Invest. 2012;72:212–20. 10.3109/00365513.2011.649015 22233479

[13] Braga F Infusino I Panteghini M. Performance criteria for combined uncertainty budget in the implementation of metrological traceability. Clin Chem Lab Med. 2015;53:905–12. 10.1515/cclm-2014-1240 25870964

[14] Korol W Rubaj J Bielecka G Walczyński S Reszko-Zygmunt J Dobrowolski R. Criteria for using proficiency test results for estimation of measurement uncertainty: feed analysis example. Accredit Qual Assur. 2017;22:83–9. 10.1007/s00769-017-1252-1

[15] Magnusson B, Näykki T, Hovind H, Krysell M, Sahlin E. Handbook for calculation of measurement uncertainty in environmental laboratories. Nordtest Report TR 537 (ed. 4) 2017. Available at www.nordtest.info. Accessed: 6 Nov 2018.

[16] Medina-Pastor P Valverde A Pihlstrom T Masselter S Gamon M Mezcua M Comparative study of the main top-down approaches for the estimation of measurement uncertainty in multiresidue analysis of pesticides in fruits and vegetables. J Agric Food Chem. 2011;59:7609–19. 10.1021/jf104060h 21155569

[17] Comité français d’accréditation. COFRAC. SH GTA 14. Guide technique d’accreditation pour l’evaluation des incertitudes de mesure en biologie medicale. Available at http://www.cofrac.fr. Accessed: 6 Nov 2018.

[18] Matar G Poggi B Meley R Bon C Chardon L Chikh K Uncertainty in measurement for 43 biochemistry, immunoassay, and hemostasis routine analytes evaluated by a method using only external quality assessment data. Clin Chem Lab Med. 2015;53:1725–36. 10.1515/cclm-2014-0942 25811667

[19] Haeckel R Wosniok W Gurr E Peil B. Permissible limits for uncertainty of measurement in laboratory medicine. Clin Chem Lab Med. 2015;53:1161–71. 10.1515/cclm-2014-0874 25720082

[20] Westgard JO. Useful measures and models for analytical quality management in medical laboratories. Clin Chem Lab Med. 2016;54:223–33. 10.1515/cclm-2015-0710 26426893

[21] Aarsand AK Roraas T Bartlett WA Coşkun A Carobene A Fernandez-Calle P Harmonization initiatives in the generation, reporting and application of biological variation data. European Federation of Clinical Chemistry and Laboratory Medicine Working Group on Biological Variation. Clin Chem Lab Med. 2018;56:1629–36. 10.1515/cclm-2018-0058 29596051

[22] Westgard JO. Quality requirements. Desirable biological variation database specifications. Desirable Specifications for Total Error, Imprecision, and Bias, derived from intra- and inter-individual biologic variation. Available at www.westgard.com/biodatabase1.htm. Accessed July 4th 2018.

[23] Ceriotti F. Deriving proper measurement uncertainty from Internal Quality Control data: An impossible mission? Clin Biochem. 2018;57:37–40. 10.1016/j.clinbiochem.2018.03.019 29605551

[24] Padoan A Antonelli G Aita A Sciacovelli L Plebani M. An approach for estimating measurement uncertainty in medical laboratories using data from long-term quality control and external quality assurance schemes. Clin Chem Lab Med. 2017;55:1696–701. 10.1515/cclm-2016-0896 28245184

[25] Dallas Jones GR. Measurement uncertainty for clinical laboratories - a revision of the concept. Clin Chem Lab Med. 2016;54:1303–7. 10.1515/cclm-2016-0311 27176746

[26] Westgard JO. Quality requirements. Consolidated Comparison of Chemistry Performance Specifications. Available at https://www.westgard.com/consolidated-goals-chemistry.htm Accessed: 04 Nov 2019.

[27] Oosterhuis WP Theodorsson E. Total error vs. measurement uncertainty: revolution or evolution? Clin Chem Lab Med. 2016;54:235–9. 10.1515/cclm-2015-0997 26540227

[28] Qin Y Zhou R Wang W Yin H Yang Y Yue Y Uncertainty evaluation in clinical chemistry, immunoassay, hematology and coagulation analytes using only external quality assessment data. Clin Chem Lab Med. 2018;56:1447–57. 10.1515/cclm-2017-1199 29683797

